# Emotion Recognition from Spatio-Temporal Representation of EEG Signals via 3D-CNN with Ensemble Learning Techniques

**DOI:** 10.3390/brainsci13040685

**Published:** 2023-04-19

**Authors:** Rajamanickam Yuvaraj, Arapan Baranwal, A. Amalin Prince, M. Murugappan, Javeed Shaikh Mohammed

**Affiliations:** 1 National Institute of Education, Nanyang Technological University, Singapore 637616, Singapore; 2 Department of Computer Science and Information Systems, BITS Pilani, Sancoale 403726, Goa, India; 3 Department of Electrical and Electronics Engineering, BITS Pilani, Sancoale 403726, Goa, India; 4 Intelligent Signal Processing (ISP) Research Lab, Department of Electronics and Communication Engineering, Kuwait College of Science and Technology, Block 4, Doha 13133, Kuwait; 5 Department of Electronics and Communication Engineering, Faculty of Engineering, Vels Institute of Sciences, Technology, and Advanced Studies, Chennai 600117, Tamilnadu, India; 6 Centre for Excellence in Unmanned Aerial Systems (CoEUAS), Universiti Malaysia Perlis, Kangar 02600, Perlis, Malaysia; 7 Department of Biomedical Technology, College of Applied Medical Sciences, Prince Sattam bin Abdulaziz University, Al Kharj 11942, Saudi Arabia

**Keywords:** hybrid models, 3D-CNN, deep neural networks, machine learning classifiers, emotion recognition

## Abstract

The recognition of emotions is one of the most challenging issues in human–computer interaction (HCI). EEG signals are widely adopted as a method for recognizing emotions because of their ease of acquisition, mobility, and convenience. Deep neural networks (DNN) have provided excellent results in emotion recognition studies. Most studies, however, use other methods to extract handcrafted features, such as Pearson correlation coefficient (PCC), Principal Component Analysis, Higuchi Fractal Dimension (HFD), etc., even though DNN is capable of generating meaningful features. Furthermore, most earlier studies largely ignored spatial information between the different channels, focusing mainly on time domain and frequency domain representations. This study utilizes a pre-trained 3D-CNN MobileNet model with transfer learning on the spatio-temporal representation of EEG signals to extract features for emotion recognition. In addition to fully connected layers, hybrid models were explored using other decision layers such as multilayer perceptron (MLP), k-nearest neighbor (KNN), extreme learning machine (ELM), XGBoost (XGB), random forest (RF), and support vector machine (SVM). Additionally, this study investigates the effects of post-processing or filtering output labels. Extensive experiments were conducted on the SJTU Emotion EEG Dataset (SEED) (three classes) and SEED-IV (four classes) datasets, and the results obtained were comparable to the state-of-the-art. Based on the conventional 3D-CNN with ELM classifier, SEED and SEED-IV datasets showed a maximum accuracy of 89.18% and 81.60%, respectively. Post-filtering improved the emotional classification performance in the hybrid 3D-CNN with ELM model for SEED and SEED-IV datasets to 90.85% and 83.71%, respectively. Accordingly, spatial-temporal features extracted from the EEG, along with ensemble classifiers, were found to be the most effective in recognizing emotions compared to state-of-the-art methods.

## 1. Introduction

An emotion is a psycho-physiological experience resulting from a conscious or unconscious perception of a situation, object, or characteristic. It is often related to mood, temperament, and personality [1]. Emotions are vital aspects of human existence and play an imperative role in our lives. The ability to understand emotions is crucial when it comes to human–computer interaction (HCI) and clinical settings. Currently, emotion recognition has attracted major attention due to its potential applications in a variety of fields. A wide range of application areas are developing very rapidly, such as virtual reality (VR), gaming, health care, marketing, e-learning, and recommendation systems [2]. These application areas use emotion recognition to interact with humans at high levels.

Many studies have been previously conducted on emotion recognition. The input modes used in these studies can generally be divided into physiological and non-physiological signals. A non-physiological signal is composed of external signals such as gestures, facial expressions, verbal tones, etc. [3]. Most modern HCI systems still lack emotional intelligence and the ability to utilize these signals. An individual’s consciousness can easily influence and control non-physiological signals, but physiological signals represent their emotional state more accurately and consistently [4,5]. There are several types of physiological signals, such as electroencephalograms (EEGs), electrooculography (EOGs), electromyography (EMGs), etc. Among the types of physiological signals, EEG signals originate from the cortex of the brain, the region believed to be largely responsible for individual thoughts, emotions, and behaviors. EEG measures electrical activity in the brain using small metal electrodes attached to the scalp [6]. With recent advances in battery technology, EEG has become a more portable, reliable, and relatively inexpensive method of monitoring brain activity compared to other methods [7].

Although EEG offers many advantages in recognizing emotions, it still has some limitations. EEG has a very low spatial resolution in comparison to its temporal resolution. Furthermore, EEG signals suffer from poor signal-to-noise ratios (SNR) [8]. Besides these limitations, EEG signals also have poor homogeneity and generalizability across participants, which has hampered cross-subject emotion recognition studies [9]. Consequently, most of the studies are aimed at developing subject-dependent systems rather than subject-independent systems. Various approaches have also been studied to overcome the limitations, which generally divide the entire emotion recognition pipeline into three stages: signal preprocessing, feature extraction, and classification.

Today, several datasets for emotion recognition using EEG signals are available, of which the most well-known are DEAP (A dataset for emotion analysis using EEG, physiological, and video signals) [10], DREAMER (A Database for Emotion Recognition through EEG and ECG Signals from Wireless Low-cost Off-the-Shelf Devices) [7], SEED (SJTU Emotion EEG Dataset with three emotion classes) [11], and SEED-IV (SJTU Emotion EEG Dataset (SEED) with four emotion classes) [11]. The SEED and SEED-IV datasets were used here as there are extensive studies on them that provide ground for comparison. Moreover, to the best of our knowledge, this is the first time that spatio-temporal features have been used in SEED and SEED-IV.

This paper aims to investigate subject-dependent emotion recognition using the SEED and SEED-IV datasets. Spatial-temporal representations of EEG signals were used as the input modality on which a pre-trained 3D-CNN model coupled with transfer learning was used for feature extraction. To preserve the spatial and temporal information in the EEG signals, the signals were preprocessed and given a 3D block representation based on electrode arrangements.

The main contributions of this work are as follows:

- A 3D-CNN model pre-trained using transfer learning was used to extract features from spatio-temporal 3D representations of EEG signals. The study used spatial information from 62 electrodes to create input modality. Using 3D-CNN and transfer learning with post-filtering, this is the first time that emotion classification has been performed on the SEED datasets based on spatiotemporal features.
- Apart from the traditional fully connected layers (used for classifying after feature extraction from CNN), other major classifiers, including k-nearest neighbor (KNN), extreme learning machine (ELM), XGBoost, and random forest in hybrid models, were also used. Furthermore, the post-filtering of output labels was studied.
- A comprehensive set of results is presented to demonstrate the accuracy and efficiency of the proposed approaches on the SEED and SEED-IV datasets. Both the individual subject’s accuracy and the average subject’s accuracy across subjects are reported. The reporting of each subject’s accuracy enhances transparency and provides a baseline against which other researchers can compare their work. The computation time to evaluate EEG signals is also reported to demonstrate the efficiency of the proposed methodologies.

## 2. Related Works

The use of deep neural networks (DNNs) for emotion recognition has received considerable attention and has achieved notable success in recent years. This section reviews previous literature on emotion identification using EEG signals based on DNNs. Using statistical features (mean, median, mode, and range) with shallow classifiers [12], 75% accuracy was achieved on the DEAP dataset using Naive Bayes, KNN, decision trees, and SVM. Qing et al. [13] achieved 74.87% accuracy on the SEED dataset and 62.63% on the DEAP dataset using an ensemble model (EM) consisting of shallow classifiers, including k-nearest neighbor (KNN), decision tree (DT), and random forest (RF), with a soft-voting strategy. Chen et al. [14] proved that the DNN-based approaches outperformed shallow classifiers in terms of performance in recognizing emotions. Tarán et al. [15] proposed using a combination of sample entropy (SampEn), Tsallis entropy (TE), Higuchi fractal dimension (HFD), and Hurst exponent (HE) with a multiclass least squares support vector machine (SVM) model for their analysis. They employed empirical mode decomposition (EMD)/intrinsic mode function (IMF) filters to clean the data, along with variational mode decomposition (VMD) filters to ensure data integrity. They achieved an accuracy of 90.63% on their dataset for four emotion classifications (happiness, sadness, fear, and neutral). Using the CNN-SAE (sparse autoencoder)-DNN model in combination with the Pearson correlation coefficient (PCC) between different channels as a feature, Liu et al. [16] achieved 96.77% accuracy on the SEED dataset, which is considered to be state-of-the-art.

To make use of the spatial information contained in EEG signals, several graph-based techniques that use the signals’ spatial information were studied. Using differential entropy (DE), power spectral density (PSD), differential asymmetric feature (DASM), rational asymmetric feature (RASM), and differential causality (DCAU), Song et al. [17] proposed a dynamic graph convolution network (DGCNN) model with handcrafted features (DE, PSD, DASM, and DCAU) to classify emotions. They achieved an accuracy of 90.4% (three emotions) on the SEED dataset. In a subsequent study by Zhang et al. [18], the same features used by Song et al. for a graph convolutional broad network model were applied, and the model’s accuracy increased to 94.24% on the SEED dataset. Zhong et al. [19] adopted regularized graph neural networks (RGNN) as a means of computing pre-computed differential entropy features of the SEED and SEED-IV datasets and achieved a state-of-the-art performance of 79.34% for the SEED-IV dataset.

Convolutional neural networks (CNNs) are neural networks with one or more convolutional layers. They are generally used for image processing, classification, segmentation, and other autocorrelated data processing [20]. As a result of their computational efficiency, CNNs are highly effective at detecting and learning important features without any intervention from humans. CNNs can be classified based on their convolutional kernel dimension. 2D CNNs use 2D convolutional kernels and utilize context across the height and width of 2D frames (spatial features) to make predictions. However, they are inherently incapable of leveraging information from adjacent frames. Three-dimensional CNNs solve this problem, as they are the 3D equivalent of two-dimensional CNNs [21].

In recent years, 3D-CNN has achieved a considerable amount of success when it comes to processing spatio-temporal information such as action recognition [1,7,22]. This capability was also used in several studies to recognize emotions using 3D-CNN. 3D convolutional kernels are capable of handling the voxel information from adjacent frames, making them powerful models for learning representations of volumetric data, such as videos and 3D medical images (MRI, CT scan) [23]. 3D-CNN has been used in several studies to effectively extract features from videos. Therefore, the current study used 3D-CNNs to extract features from the 3D spatio-temporal representation of EEG data [6,24,25]. Salama et al. [24] used a 3D-CNN model to classify emotions, and a 3D representation of the data was created for the inputs of the model. They achieved an accuracy of 87.44% for arousal (two classes) and 88.49% for valence (two classes) on the DEAP dataset. A study by Cho et al. [6] used two different end-to-end 3D-CNN architectures, C3D and R(2) + 1D, to extract features in their study. They achieved an accuracy of 99.73% (4 classes) on the DEAP dataset. The authors proposed the use of a novel method to represent EEG signals in a 3D spatio-temporal block by setting the position of the channels at the sampling time at their original positions, after which the interpolation of 2D EEG frames was used to reconstruct the 3D signals. A transfer learning method was used by Cimtay et al. [26] to recognize emotions based on a pre-trained Inception-Resnet-V2 model. They achieved a cross-subject accuracy of 86.56% for two classes (positive-negative) and 78.34% for three classes (positive-neutral-negative) on the SEED dataset. In [25], EEG data were extracted from three international open-source datasets, such as DREAMER, SEED, and DEAP, to classify emotions using 3D-CNN. The maximum mean classification rate was 97.64% using SEED datasets.

Most earlier studies did not use the spatial information between the adjacent electrodes, which, according to some studies, is an important aspect of the input. Unlike most studies that have handcrafted features using different techniques like Pearson correlation coefficient (PCC), Principal Component Analysis (PCA), Higuchi Fractal Dimension (HFD), entropy studies, etc., for the input of the DNN, the current study created a simple 3D representation using the raw EEG signals, which preserves both the spatial and temporal information of the data. Additionally, this study completely relied on DNN’s ability to extract meaningful features that could be fed into the classifier for the classification of emotions. Furthermore, this work used a pre-trained 3D-CNN model with transfer learning as the DNN model in the study. Earlier studies on SEED datasets that used 3D CNNs did not take spatial information into account, alongside the transfer learning approach, in emotion recognition. Some studies have used graph-based neural networks that use spatial information. The novelty of this paper is the use of 3D CNNs with spatio-temporal features to recognize emotion on SEED datasets. In the study, the spatial information from 62 electrodes was used to create input modality. As far as we know, this is the first time that transfer learning has been used on SEED datasets to classify emotions using spatio-temporal features with 3D-CNN and an ensemble classifier.

## 3. Dataset

The experiments were conducted using two datasets developed by SJTU: SEED and SEED-IV. The SEED dataset is classified into three categories (positive, negative, and neutral), and the SEED-IV dataset is classified into four categories (happiness, sadness, fear, and neutral). The average score was calculated for each subject using subject-dependent classification, i.e., each model was trained and tested individually for each subject. No cross-validation was performed since average scores were reported across all subjects. Phased evaluations of 3D MobileNet models were conducted for each of the 15 subjects in an 80:20 split ratio.

### 3.1. SEED

A multimodal dataset called SEED [11] was developed by researchers in the Brain-like Computing and Machine Intelligence (BCMI) laboratory at Shanghai Jiao Tong University (SJTU). It consisted of EEG signals that were collected from 15 subjects (7 males and 8 females; mean age: 23.27 years; standard deviation: ±2.37). An ESI NeuroScan System with 62 channels was used to record the subjects’ responses in response to 15 clips (around 4 min long) of a Chinese film, which were viewed by the subjects on their watches. The film clips were carefully selected to evoke a range of emotions, such as positive, neutral, and negative ones. Each subject experimented three times, with an interval of approximately one week between each repetition. Finally, the data were downsampled from 1000 to 200 Hz sampling rates, and a band-pass frequency filter of 0–75 Hz was applied to remove noise and artifacts.

### 3.2. SEED-IV

As a multimodal dataset, SEED-IV [11] contains the EEG signals of 15 subjects, ranging in age from 20 to 24 years old, with 7 males and 8 females. A 62-channel ESI NeuroScan System was used to record EEG signals while subjects watched film clips. Seventy-two short film clips of about two minutes each were used to induce four target emotions: happiness, sadness, fear, or neutrality. The experiments were conducted in three sessions with 24 trials per session (6 trials per emotion) on different days. Initially, the data were collected at a sampling frequency of 1000 Hz, but the sample rate was later downscaled to a sampling frequency of 200 Hz. The EEG dataset was processed with a bandpass filter between 1–75 Hz to filter out the noise and remove artifacts.

## 4. Proposed Approach

### 4.1. Spatio-Temporal Representation of EEG

SEED and SEED-IV datasets were acquired using the 62-channel ESI NeuroScan System to record the participant’s EEG signals. A one-dimensional signal (Amplitude vs. time) was recorded by the system. In a film clip of T s, 200 × T samples were collected for each of the 62 electrodes, with a sampling rate of 200 Hz.

A one-dimensional vector can be used to represent the electrode used to acquire the EEG signals at a certain time (where *t* is the time in seconds, and t=0,1,…,N−1), and *N* is the total number of samples:

*ν* = [*c*^1^*t*, *c*^2^*t*, *c*^3^*t*, …, *c*^61^*t*, *c*^62^*t*] (1)

where *c*^n^t is the recording of the nth channel at the timestamp t.

The entire data can be represented by stacking such 1D vectors into a 2D vector of dimension 62 × N as:

*C* = [*ν*_0_, *ν*, *ν*_2_, …, *ν*_N−2_, *ν*_N−1_] (2)

Many of the earlier studies [27,28,29] have used this 2D vector representation of EEG signals, which, although containing temporal information, lacks spatial information and the spatial distribution of electrodes. For these studies, information about adjacent channels and symmetrical channels is ignored. Multiple studies [5,6] have highlighted the importance of this information.

Some works [5,6] have presented an imperfect representation of EEG signals that maps electrode arrangements on the skull to a 2D plane. The method has been used for 14-channel (DREAMER) and 32-channel (DEAP) EEG datasets [5], which, in the current study, was extended to 62-channel SEED and SEED-IV datasets.

**Figure 1:**
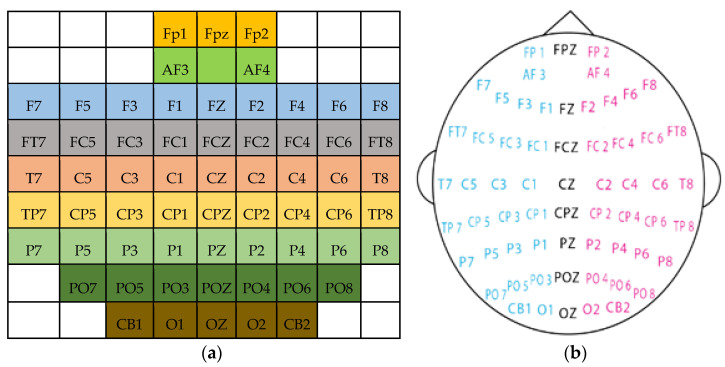
(**a**) EEG electrode arrangements on 2D 9 × 9 frame; (**b**) Electrode placement in SEED and SEED-IV datasets. Schemes follow another format [29,30,31].

As shown in Figure 1, channels were arranged in a 9 × 9 matrix, which represents spatial information. It is noteworthy that this representation does not accurately portray the actual arrangement, so the spatial information is slightly distorted.

**Figure 2:**
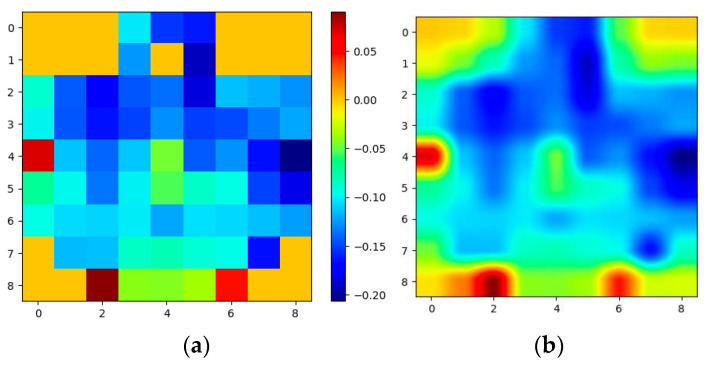
Illustration of spatio-temporal conversion: (**a**) 2D representation of raw EEG signals at time t; (**b**) 2D representation of EEG signals after interpolation at time t.

In the 2D sparse representation, all the empty boxes indicate that the corresponding electrodes are absent. The empty boxes were filled using interpolation to make the representation dense. Interpolation was performed using radial basis functions (RBFs) and Gaussian basis functions [6]. A simple illustration of the 2D representation of EEG signals, the 2D-EEG signals after time interpolation, and the 3D-EEG representation is shown in Figure 2.

The 3D EEG stream, S, is then created by stacking the 2D frames one after another.

*S* = [*f*_t_, *f*_+1_, *f*_+2_, …, *f*_t+w−1_] (3)

where *f*_t_ is the 2D frame at timestamp t and w is the length of the time window.

**Figure 3:**
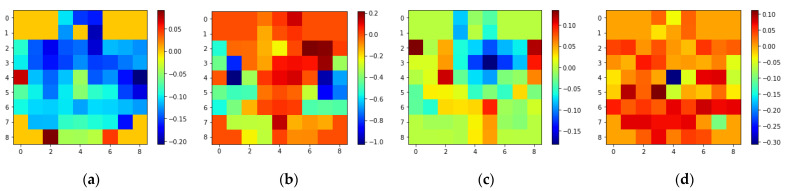
2D 9 × 9 EEG frame samples of different emotions of SEED-IV: (**a**) Happiness; (**b**) Sadness; (**c**) Fear; (**d**) Neutral.

**Figure 4:**
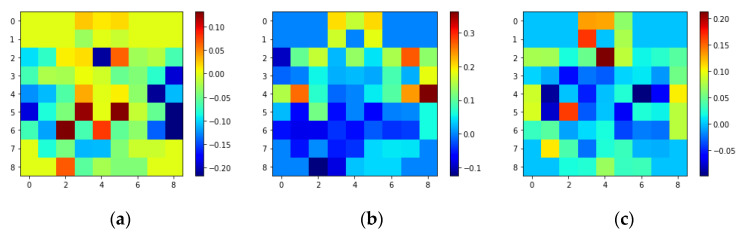
2D 9 × 9 EEG frame samples of different emotions of SEED: (**a**) Positive; (**b**) Neutral; (**c**) Negative.

Based on previous studies, one second was determined to be an appropriate time window for recognizing emotions [6]. As a result, w (sampling frequency) was set to 200. Before concatenating into 3D EEG streams, the 2D frames were resized from 9 × 9 to 64 × 64 to make spatial dimensions comparable to temporal dimensions. Figure 3 and Figure 4 show the average of all the subjects’ 2D representations of different emotions in the SEED-IV and SEED datasets, respectively.

### 4.2. Spatio-Temporal Learning Based on 3D-CNNs

A resource-efficient 3D-CNN network was used for emotion recognition in this study. Based on the well-known resource-efficient 2D CNNs, 3D resource-efficient CNNs were developed [32]. Several portable, wearable, wireless, low-cost, off-the-shelf devices are available on the market today that allow for effective computing methods to be utilized in everyday life. As lightweight networks, resource-efficient models are ideal for mobile and embedded applications since they can be integrated with portable, wearable, EEG devices. This work includes testing the following pre-trained models: 3D-Mobile Net, 3D-ShuffleNet, 3D-MobileNetv2, 3D-ShuffleNetv2, and 3D-EfficientNet for emotion recognition (available at https://github.com/okankop/Efficient-3DCNNs (accessed on 3 January 2023). Among these models, 3D-MobileNet reported the highest accuracy and the lowest computational complexity [33]. Therefore, we implemented 3D-MobileNet-based transfer learning in SEED datasets to recognize emotions.

**Figure 5:**
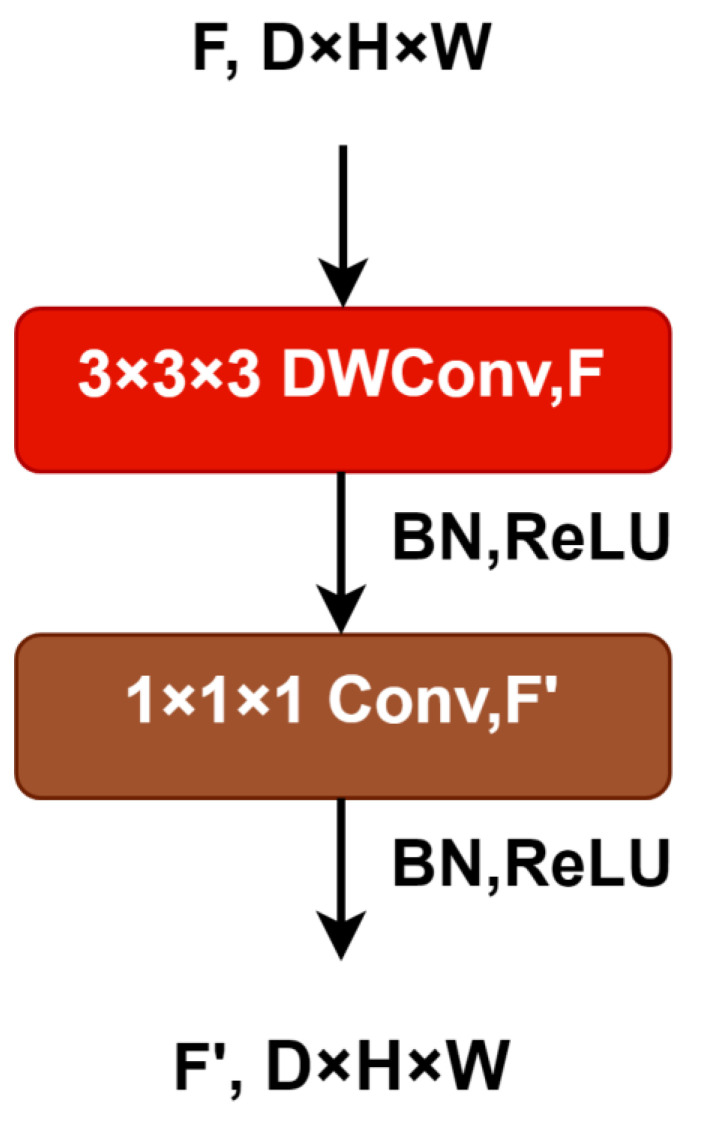
MobileNet Block (D × H × W is depth × height × width, BN: Batch normalization, ReLU: Rectified Liner Unit activation function).

The use of transfer learning is well-studied. Transfer learning is the process of improving learning for a new task by transferring knowledge from a related task that has already been learned [34]. In the past, there have been several studies that have used transfer learning to recognize emotions. A study conducted by Feng K and Chaspari T (2020) found that transfer learning can be applied to speech, video, and images, and to physiological signals related to emotion [35]. According to [26], Cimtay et al. employed a state-of-the-art pre-trained InceptionResNet model and achieved excellent performance on the SEED and DEAP datasets. In this study, 3D MobileNet was pre-trained on the Jester dataset for transfer learning. The Jester gesture recognition dataset contains labeled video clips of humans performing basic, predefined hand gestures in front of a webcam or laptop camera [22]. The 3D MobileNet network was further enhanced by the addition of dense layers (fully connected layers) to provide greater depth and accuracy when classifying complex data [26]. The weights of pre-trained 3D MobileNet models were frozen, and only the dense layer weights were trained in transfer learning. As a result, the model was optimized to prevent overfitting and reduce computation time. The description of different blocks of the proposed 3D-MobileNet architecture is given in Table 1, and the MobileNet block is shown in Figure 5. The training parameters of the proposed 3D-MobileNet are specified in Table 2. In Table 1, the input clip, due to the fact that the 3D MobileNet was trained on RGB datasets, we concatenated the 1 × 200 (temporal) × 64 × 64 (spatial) data three times to reach the required three channels.

ArgMax selects the label with the greatest probability in the last dense layer, which consists of neurons equal to the number of output classes. In the model, the ReLu activation function was used. ReLU is rectified linear unit activation function, and its abbreviation is already included in Figure 5. Mathematically, it can be defined as g(z) = max {0, z}. Due to its superior performance and ease of training, ReLu has become the default activation function for many neural networks. Additionally, the softmax activation function was used in the last dense layer to calculate the class probabilities. Table 3 shows the detailed specification of the fully connected layer in the proposed model.

**Figure 6:**
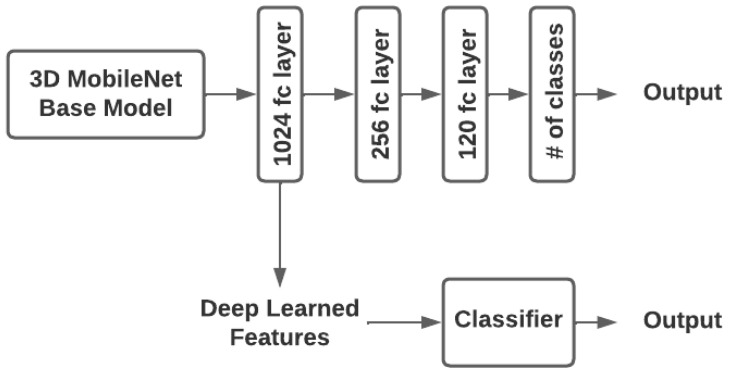
MobileNet Block (fc: Fully connected layer, # number).

After training the CNN model with the multiple-layer perceptron (MLP) classifier, the model was used to extract features. Deep-learning features were collected using a dense layer-1 (1024 neurons). Those features were then fed into other classifiers as input, which were then trained. In this study, k-nearest neighbor (KNN), Support Vector Machine (SVM), Extreme Gradient Boosting (XGB), Random Forest (RF), and Extreme Learning Machine (ELM) were used as classifiers (decision layers) (Figure 6).

- **SVM:** A supervised machine learning algorithm used for classification. The SVM algorithm seeks to find an N-dimensional hyperplane that distinctly classifies the data points.
- **XGB:** This machine learning library implements gradient-boosted decision trees (GBDT) that are scalable and distributed. This machine-learning library performs regression, classification, and ranking using parallel tree boosting.
- **RF:** It is a supervised machine learning algorithm that is widely used in classification and regression. A decision tree is constructed from different samples, the majority vote is taken for classification, and the average is taken for regression.
- **kNN:** It is one of the simplest machine learning algorithms based on the supervised learning technique. The method uses proximity to classify or predict the grouping of an individual data point.
- **ELM:** They are feedforward neural networks with one hidden layer capable of learning more quickly than gradient-based methods.

A grid search was employed to determine the best hyperparameters for the classifiers. The results for all the above-discussed classifiers are presented in this study.

### 4.3. Post-Filtering on Output Classes

Due to the high sensitivity of EEG to noise, it is very important to filter the signals prior to use. In previous studies on medical diagnostics, EEG recordings were smoothed using various filters, such as median filters, mode filter, mean filter, smooth filters, etc. Emotions are intense feelings that last for a short period [27]. Emotions are mental states that affect physiological and psychological feelings. They have a natural beginning, a natural lifespan, and a natural end. Approximately 90 s is the average duration of emotion in the human brain, according to modern neurology [28]. It is reasonable to assume that the emotions in a healthy individual with effective emotional regulation will remain constant (or not change) for some small interval T. A prior study proposed post-filtering for output classes based on this assumption [28]. The post-filtering process in this study is similar to processes used in previous studies.

**Figure 7:**
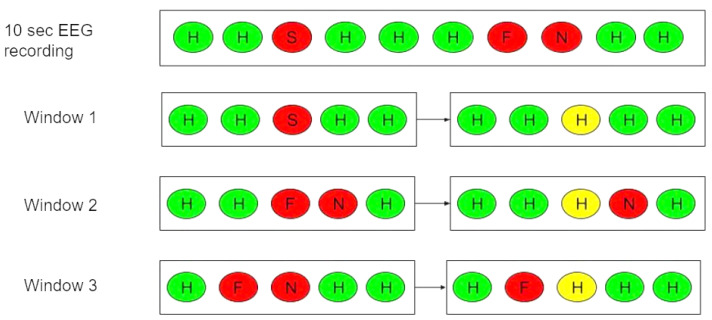
Post-filtering on 10 s output labels with post-filtering of 5 s window size.

Figure 7 shows the EEG recording for 10 s and the post-filtering process. Nevertheless, the window size is only five seconds. We illustrated the window size of five seconds working on a 10 s EEG clip for illustration purposes. A study conducted by [26] suggests that the emotional state remains the same for some short time interval T. They used a post-filtering window size of six seconds for their analysis. A mode filter with a 5 s window size was applied to the 10 s output labels. Models that predict incorrect labels are highlighted in red. Labels predicted correctly by the model are green. A yellow label indicates that the mode filter has changed the label. Using the model, the third label in window 1 was predicted to be sadness (S). Based on the model, happiness (H) was predicted to be the mode of emotions, so sadness (S) could be changed to happiness (H). Likewise, for Windows 2 and 3, we could change the fear (F) and neutral (N) predictions in the respective windows to happiness (H) predictions. Here, the window shifts by one second each time.

### 4.4. Performance Assessment

The objective of this study was to recognize emotions based on the subject’s emotions. To maintain the balance of the dataset, the EEG recordings were split into an 80:20 training–test ratio for each subject. All 15 subjects were trained individually with the models. The seed value was fixed to make a fair comparison between the models and to make the results reproducible. The trained model was applied to the test dataset after training. The performance of CNN-based hybrid models was evaluated using the following metrics.

(1) Accuracy = (*TP* + *TN*)/(*TP* + *FP* + *FN* + *TN*) (4)

(2) Precision = *TP*/(*TP* + *FP*) (5)

(3) Recall (Sensitivity) = *TP*/(*TP* + *FN*) (6)

(4) F1 Score = 2(*RecCal* ∗ *Precision*)/(*Recall* + *Percision*) (7)

where *TP* = true positive, *TN* = true negative, *FP* = false positive, and *FN* = false negative The results reported were averaged across all subjects.

## 5. Experimental Results and Discussion

This section summarizes the main findings of this study on emotion recognition using EEG signals for 3D-CNN MobileNet-based models. PyTorch was used to implement 3D MobileNet. An Intel^®^ CoreTM *i9*-10920X CPU running at 3.50 GHz and an Nvidia Corporation 2204 GPU running Ubuntu was used for the experiment. Accuracy and cross-entropy loss values were used to evaluate the convergence of the model. The model weights were saved at the point of convergence, i.e., the least loss value, to prevent overfitting.

### 5.1. 3D-CNN MobileNet Model with MLP Classifier (Traditional 3D-CNN Model)

Three-dimensional CNN models with MLP classifiers were trained for each subject. After training the model, it was evaluated on a test dataset. For both datasets, the CNN-MLP model produced results comparable to the state-of-the-art. The accuracy for the SEED-IV dataset was 78.32%, and the accuracy for the SEED dataset was 88.58% for 15 subjects (Table 4a and Table 5a). Compared to the MLP classifier, the ELM reported higher accuracy for both datasets compared to the other classifiers.

### 5.2. 3D-CNN MobileNet Hybrid Model

This phase involves extracting features from the trained models from Phase 1. The extracted features were then used to train different classifiers such as SVM, random forest, XG boost, KNN, and extreme learning machine. Hyperparameters for classifiers were optimized using the grid search technique. Compared to the CNN-MLP model, the CNN hybrid model showed significant improvement. However, the CNN-ELM hybrid model performed better, with 81.60% accuracy for the SEED-IV dataset (Table 4a) and 89.18% accuracy for the SEED dataset (Table 5a). The confusion matrices of the best classifier output is shown in Table 4b and Table 5b for SEED-IV, and SEED dataset, respectively.

### 5.3. 3D-CNN Model with Post-Filtering

**Figure 8:**
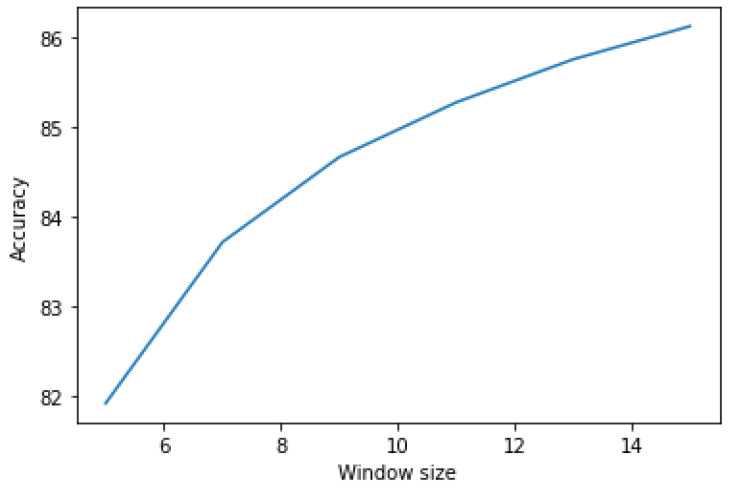
Trend chart showing an increase in accuracy (in %) with an increase in window size.

In this phase, the output labels from phases 1 and 2 were post-filtered using mode filters. Table 6 shows the results after applying post-filtering to the labels. Various window sizes from 5 to 15 s were used for post-filtering. Increasing the window size resulted in a significant increase in average accuracy (Figure 8). Due to post-filtering, the mispredictions were corrected by changing the prediction with the window mode emotion. The performance of the proposed 3D-CNN with ELM is shown in Table 7 and Table 8 using the SEED-IV and SEED datasets, respectively. In Table 8, only subject 6 reported a lower accuracy in recognizing emotions among 15 subjects. There may be a few reasons as to why subject 6 does not perform well. Some of the reasons could be (a) the signals might be corrupted by noises and other external interferences, (b) the subject is not cooperating during the experiment, or that (c) the subject might have already participated in a similar kind of experiment and that bias might affect the model. 

Table 9 compares the results of the current study with earlier studies on the SEED and SEED-IV datasets. Many earlier studies have used graph neural networks and recurrent neural networks (RNNs) to classify emotions using SEED or DREAMER datasets [17,18,29,36]. To classify emotions, the researchers have introduced a broad learning system method in graph convolutional neural networks [29]. Researchers have also studied different numbers of EEG channels for detecting emotions in order to reduce the complexity of the emotion recognition system. It was found that systems with fewer EEG channels are more accurate at detecting emotions than systems with a greater number of channels. Spatio-temporal information was recently used to classify emotions using facial expression data and EEG. Subject-specific and subject-independent emotion classifications have been conducted by some researchers using public databases [17]. Using spatio-temporal features, we were not able to find any research that used transfer learning or ensemble classifiers to recognize emotion. The comparable state-of-the-art results of this study demonstrate the capability of the 3D-CNN model to extract and learn spatio-temporal information from EEG signals. Additionally, it shows that pre-trained 3D MobileNet models with transfer learning can extract features that can be fed into ELM and MLP classifiers.

Based on the computation time required to evaluate one minute of EEG data at a 200 Hz sampling frequency, Table 10 and Table 11 illustrate the performance of the proposed CNN models. This time includes the time it takes to load the EEG data, load the CNN model, preprocess the dataset, and extract and evaluate features. Table 10 and Table 11 show the averages across five trials to eliminate discrepancies. During one minute of EEG recording, the proposed model recognized emotions in 8–8.5 s.

In the case of real-time implementation of emotion recognition using EEG signals, some of the parameters listed below could be considered for accurate and robust emotion detection:

- **Latency:** An emotion recognition system (ERS) must be highly responsive and recognize the user’s emotions without delay. A high latency may hamper ERS performance and effectiveness in real-time applications.
- **Precision/Accuracy:** To identify a particular emotion, it is imperative that the ERS is sufficiently accurate to distinguish between different users’ emotions. It is also a necessity to improve the user experience by making the ERS as accurate as possible.
- **Adaptability and robustness:** An emotion recognition system must adapt to identify different users’ emotions. ERS performance should also not be affected by any external or internal noise.
- **User-Friendly:** The ERS should be easy to use, and it needs to be convenient to configure it so that it recognizes the emotions of different users depending on their environment. If the setup is bulky and inconvenient to carry, people may not like to use it.

Moreover, researchers face some challenges when developing real-time emotion recognition systems for human–computer interactive devices:

- **Data Processing:** In real-time applications, efficient algorithms, and hardware are required to process data in real-time.
- **Generalizability:** The model should be robust and capable of delivering high performance to new users without prior knowledge.
- **Integration:** The integration of emotion recognition models into HCI architecture remains a major challenge. A seamless and efficient method for feeding data from the EEG setup into the model is necessary in the case of EEG-based emotion recognition systems.

It is important to note that this study has a few limitations. For example, this study uses only 3D Efficient MobileNet pre-trained models with transfer learning. Other pre-trained 3D-CNN models could be explored for better results. Additionally, only subject-dependent emotion recognition was investigated here, while subject-independent emotion recognition remains a challenge. Furthermore, future works can delve into the study of the extracted features and their explainability.

## 6. Conclusions

This study adopted the success of 3D-CNNs in video analysis, owing to their capacity to extract and learn temporal features in addition to spatial features. Using 3D-CNN, the EEG signals are represented in 3D spatio-temporal space by first converting the 1D raw EEG streams into 2D spatial streams and then stacking the 2D spatial streams into 3D EEG block streams. A 3D MobileNet network with transfer learning was used to extract and learn features from 3D EEG blocks. Additional pools and dense layers were added to the CNN network to enhance classification capabilities. In the SEED-IV dataset, four classes of samples were classified: happiness, sadness, fear, and neutral, with an accuracy of 78.32%. The SEED dataset showed an accuracy of 88.58% for classifying the samples into three groups: positive, neutral, and negative.

Additionally, the performance of hybrid models that were fed the extracted features from the 3D-CNN network into different classifiers (XG boost, random forest, support vector machine, k-nearest neighbor, and extreme learning machine), in addition to the MLP classifier (dense layers and pool layers), was examined. Compared to SEED-IV and SEED, 3D-CNN-ELM hybrid models delivered significant improvements in performance with an accuracy of 81.60% and 89.18%, respectively. The emotions of a healthy person vary very little, so it could be assumed that emotions will remain constant for a short period. These studies were used to investigate the model’s performance when post-filtering the output labels with mode filters. A time window ranging from 5 to 15 s was selected. The accuracy of the model increased as the time window of the mode filter increased. A CNN-ELM hybrid model that applied post-filtering to a 15-s window achieved an accuracy of 87.50%. The proposed model could be used for emotion recognition in HCI-related fields, healthcare, etc.

## Figures and Tables

**Table 1:** 3D-MobileNet architecture.

Layer/Stride	Repeat	Output Size
Input Clip		3 × 200 × 64 × 64
Conv(3 × 3 × 3)/s(1,2,2)	1	32 × 200 × 32 × 32
Block/s(2,2,2)	1	64 × 100 × 16 × 16
Block/s(2,2,2)	2	128 × 50 × 8 × 8
Block/s(2,2,2)	2	256 × 25 × 4 × 4
Block/s(2,2,2)	6	512 × 13 × 2 × 2
Block/s(1,1,1)	2	1024 × 13 × 2 × 2
AvgPool(13,2,2)/s(1,1,1)	1	1024 × 1 × 1 × 1

**Table 2:** Properties of layers following the 3D MobileNet base model.

Parameters	Settings
Base Model	3D MobileNet
No. of additional layers	4 Dense Layers
Optimizer	Adam
Loss function	Cross Entropy Loss
Max number of epochs	200
Max number of iterations	400,000
Shuffle	True
Batch size	4
Learning Rate	1 × 10^−4^
Train-test split ratio	80:20
Environment	Processor: Intel^®^ Core™ i9-10920X CPU @ 3.50 GHz × 24, GPU: Nvidia Corporation 2204, Pytorch
Number of classes	3 (positive, neutral, negative for SEED) and 4 (happiness, sadness, fear, neutral for SEED-IV)

**Table 3:** Fully connected layer specification for the model.

Layer (Type)	Output Shape	Connected to	Activation Function
Dense 1	(None, 1024)	convolution	-
Dense 2	(None, 256)	Dense 1	ReLu
Dense 3	(None, 120)	Dense 2	ReLu
Dense 4	(None, # of classes)	Dense 3	Softmax

**Table 4:** (**a**)**.** SEED-IV (4 classes) results for CNN hybrid models. (**b**). Confusion matrix of 3D-CNN and ELM classifier for SEED-IV dataset.

(a)					
Model	Accuracy (%)	Std. Dev (%)	F1	Precision	Recall
MLP	78.32	12.37	0.78	0.79	0.78
SVM	80.8	12.87	0.81	0.81	0.81
Random Forest	79.77	12.41	0.8	0.8	0.8
XGB	80.32	12.65	0.8	0.8	0.8
kNN	81.29	12.36	0.81	0.81	0.81
**ELM**	**81.6**	**12.04**	**0.82**	**0.82**	**0.82**
**(b)**					
	**Predicted Values**				
**Actual Values**		**Neutral**	**Sad**	**Fear**	**Happy**
	Neutral	**450**	39	23	37
	Sad	49	**451**	37	14
	Fear	30	31	**408**	29
	Happy	19	29	28	**352**

**Table 5:** (**a**). SEED (3 classes) results for CNN hybrid models. (**b**). Confusion matrix of 3D-CNN and ELM classifier for SEED dataset.

(a)					
Model	Accuracy (%)	Std. Dev (%)	F1	Precision	Recall
MLP	88.58	14.85	0.89	0.89	0.89
SVM	88.75	14.73	0.89	0.89	0.89
Random Forest	88.64	15.05	0.89	0.89	0.89
XGB	88.76	14.74	0.89	0.89	0.89
kNN	89.05	14.75	0.89	0.89	0.89
**ELM**	**89.18**	**14.72**	**0.89**	**0.89**	**0.89**
(**b**)					
		**Predicted Values**			
**Actual Values**			Positive	Neutral	Negative
		Positive	**326**	21	19
		Neutral	21	**307**	17
		Negative	18	21	**315**

**Table 6:** Post-filtering results for the SEED-IV dataset.

Dataset	SEED-IV			SEED		
Post-Filter Window	CNN-MLP (%)	CNN-kNN (%)	CNN-ELM (%)	CNN-MLP (%)	CNN-kNN (%)	CNN-ELM (%)
-	78.32	81.29	81.6	88.58	89.05	89.18
5	81.92	84.12	83.71	90.43	90.77	90.85
7	83.72	85.64	85.32	91.16	91.46	91.50
9	84.67	86.41	86.13	91.55	91.81	91.87
11	85.28	86.92	86.65	91.79	91.94	92.03
13	85.76	87.36	87.11	91.93	92.01	92.15
15	86.13	87.72	87.50	92.08	92.13	92.23

**Table 7:** Subject-wise results for SEED-IV dataset (4 classes) using 3D-CNN + ELM model.

Subject	Accuracy (%)	Precision	Recall	F1-Score
1	77.22	0.77	0.77	0.77
2	59.65	0.6	0.6	0.6
3	84.78	0.85	0.85	0.85
4	91.45	0.91	0.91	0.91
5	60.16	0.6	0.6	0.6
6	86.17	0.86	0.86	0.86
7	87.70	0.88	0.88	0.88
8	85.67	0.86	0.86	0.86
9	78.67	0.79	0.79	0.79
10	85.97	0.86	0.86	0.86
11	75.79	0.76	0.76	0.76
12	75.35	0.75	0.75	0.75
13	85.04	0.85	0.85	0.85
14	93.33	0.93	0.93	0.93
15	97.18	0.97	0.97	0.97
**Average**	**81.60**	**0.82**	**0.82**	**0.82**

**Table 8:** Subject-wise results for SEED dataset (3 classes) using 3D-CNN + ELM model.

Subject	Accuracy (%)	Precision	Recall	F1-Score
1	85.25	0.85	0.85	0.85
2	87.61	0.88	0.88	0.88
3	93.95	0.94	0.94	0.94
4	88.94	0.89	0.89	0.89
5	94.84	0.95	0.95	0.95
6	38.20	0.38	0.38	0.38
7	85.84	0.86	0.86	0.86
8	97.05	0.97	0.97	0.97
9	91.45	0.91	0.91	0.91
10	96.76	0.97	0.97	0.97
11	94.54	0.95	0.95	0.95
12	94.10	0.94	0.94	0.94
13	94.40	0.94	0.94	0.94
14	94.99	0.95	0.95	0.95
15	99.85	0.99	0.99	0.99
**Avg. with subject 6**	**89.18**	**0.89**	**0.89**	**0.89**
**Avg. w/o subject 6**	**92.83**	**92.79**	**92.79**	**92.79**

**Table 9:** Performance comparison between different relevant studies on SEED and SEED-IV datasets. The numbers in the table are the best accuracy that the study achieved.

Methods of Emotion Recognition	SEED	SEED-IV
SVM	83.99/09.92	56.61/20.05
GSCCA [29]	82.96/09.95	69.08/16.66
DBN [34]	86.08/08.34	66.77/07.38
STRNN [18]	89.50/07.63	-
DGCNN [17]	90.40/08.49	69.88/16.29
BiDANN [37]	92.38/07.04	70.29/12.63
EmotionMeter [31]	-	70.58/17.01
BiHDM [38]	93.12/06.06	74.35/14.09
RGNN [19]	94.24/05.95	79.37/10.54
3D-CNN with PST-Attention [39]	95.76/04.98	82.73/08.96
EeT (S+T Attention) [40]	96.20/04.39	83.27/08.37
**3D-CNN + MLP**	**88.58/14.85**	**78.32/12.37**
**3D-CNN + ELM**	**89.18/14.72**	**81.6/12.04**
**3D-CNN + MLP + Postfilter (5 s)**	**90.43/14.49**	**81.92/12.18**
**3D-CNN + ELM + Postfilter (5 s)**	**90.85/14.45**	**83.71/11.92**

**Table 10:** Computation time in seconds for evaluating 1 min of 62-channel EEG recording at 200 Hz sampling frequency.

Task	SEED-IV Computation Time (s)	SEED Computation Time (s)
EEG (1-min) loading from hard disk	0.968	0.945
CNN model loading from hard disk	0.053	0.051
Preprocessing (including RBF interpolation and creation of 3D blocks)	3.567	3.723
Deep-feature extraction from CNN model (GPU)	2.911	2.768
Evaluation using MLP model (GPU)	0.638	0.594

**Table 11:** Evaluation time of different classifiers (in s).

MLP	RF	ELM	SVM	kNN	XGB
0.638	0.476	0.339	0.846	0.192	0.563

## Data Availability

This work used publicly available datasets collected from SEED dataset [11].
